# Case report: MRI-negative myelitis following COVID-19 with SEP abnormalities: a case series and literature review

**DOI:** 10.3389/fneur.2023.1275696

**Published:** 2023-10-19

**Authors:** Kentaro Kawama, Rui Shimazaki, Yoko Sunami, Natsuki Miyakoshi, Shinsuke Tobisawa, Toshio Shimizu, Kazushi Takahashi

**Affiliations:** Department of Neurology, Tokyo Metropolitan Neurological Hospital, Tokyo, Japan

**Keywords:** COVID-19, myelitis, SEPs, MRI, microglia

## Abstract

Coronavirus Disease 2019 (COVID-19) is known to have various, neurological manifestations. We herein report three patients with MRI-negative myelitis following COVID-19 with abnormal somatosensory evoked potentials (SEPs). Decreased amplitude of the cortical potential and prolonged latency in the SEPs contributed to diagnosing myelitis in the present patients. The SEP findings improved as the neurological symptoms improved. Despite a delay in initiating immunosuppressive treatment after myelitis onset, all the patients improved clinically. In the light of recent progress in COVID-19 research, several hypotheses can be made to explain the pathophysiology underlying MRI-negative myelitis, including antibody-binding and microglial synapse elimination.

## Introduction

Coronavirus Disease 2019 (COVID-19), caused by severe acute respiratory syndrome Coronavirus-2 (SARS-CoV-2) infection, can have various, neurological manifestations. A review of recent studies found that neurological complications of COVID-19 may include headache, anosmia, ageusia, cerebrovascular events, encephalopathy, seizures, cerebellar ataxia, myoclonus, myopathy, myelitis, and Guillain-Barré syndrome (1) Several pathophysiological mechanisms, including autoimmunity, latent virus reactivation (2), micro-clots (3), mitochondrial dysfunction (4), and microglial activation (5), have been proposed to explain long-term, neurological complications. Herein, we describe three cases of MRI-negative myelitis following SARS-CoV-2 infection with abnormal somatosensory evoked potentials (SEPs) and present a review of recent studies of COVID-19-related myelitis.

## Case 1

**Figure 1:**
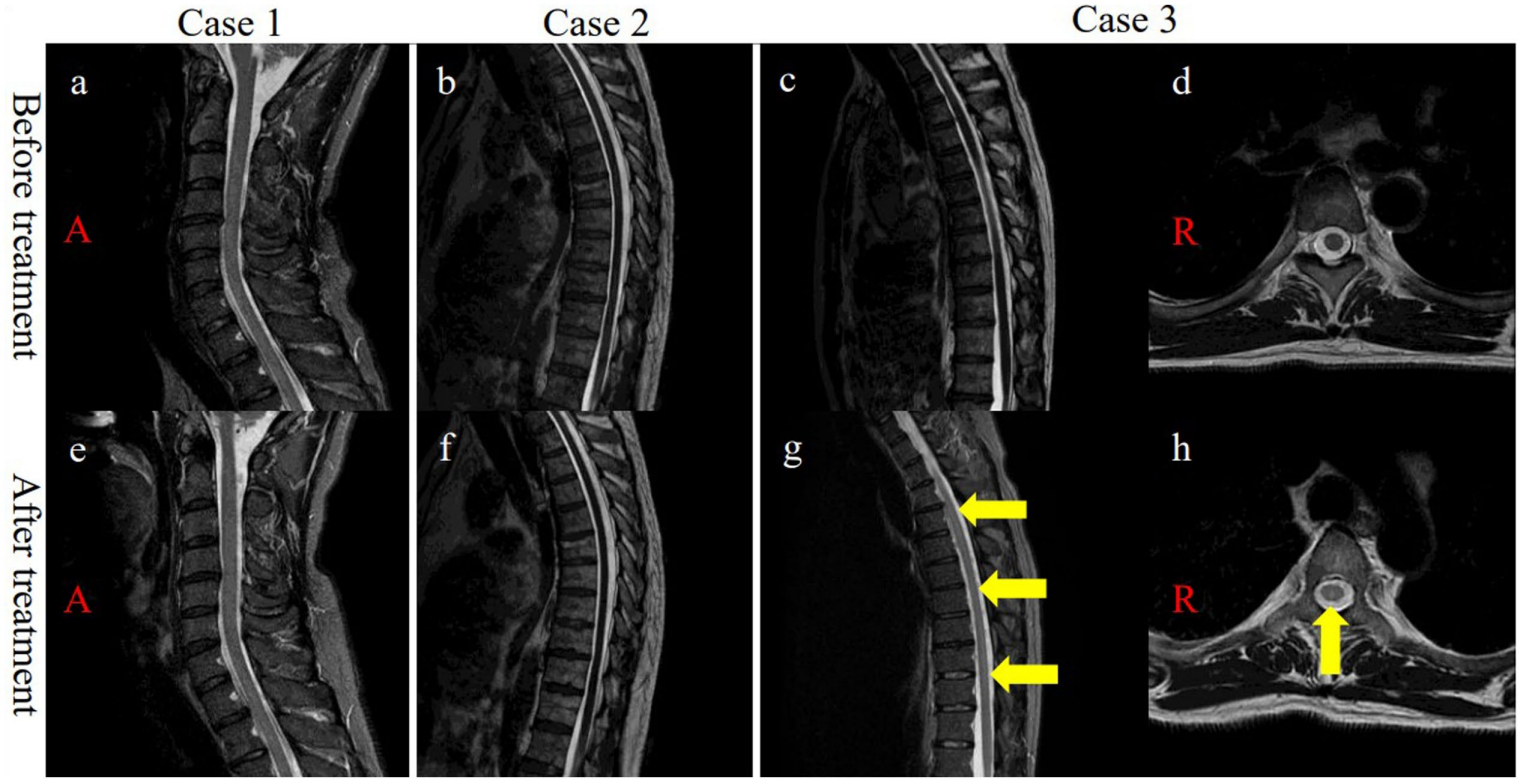
**(A)** Cervical and **(B,C)** thoracic initial MRI displayed no signal changes on sagittal T2WI. **(D)** Axial T2WI of T5 showing no signal changes. **(E)** No signal abnormalities were observed in the cervical cord after treatment. **(F)** No signal changes were observed in the thoracic cord after treatment. **(G)** Post-treatment MRI demonstrated signal changes in the dorsal columns (T1-T10) on sagittal STIR (yellow arrows, g). **(H)** Post-treatment MRI confirmed signal changes in the thoracic dorsal columns (T5) on axial T2WI (yellow arrow, h). MRI, magnetic resonance imaging; T, thoracic vertebrae, T2WI, T2-weighetd imaging; STIR, short T1 inversion recovery.

**Figure 2:**
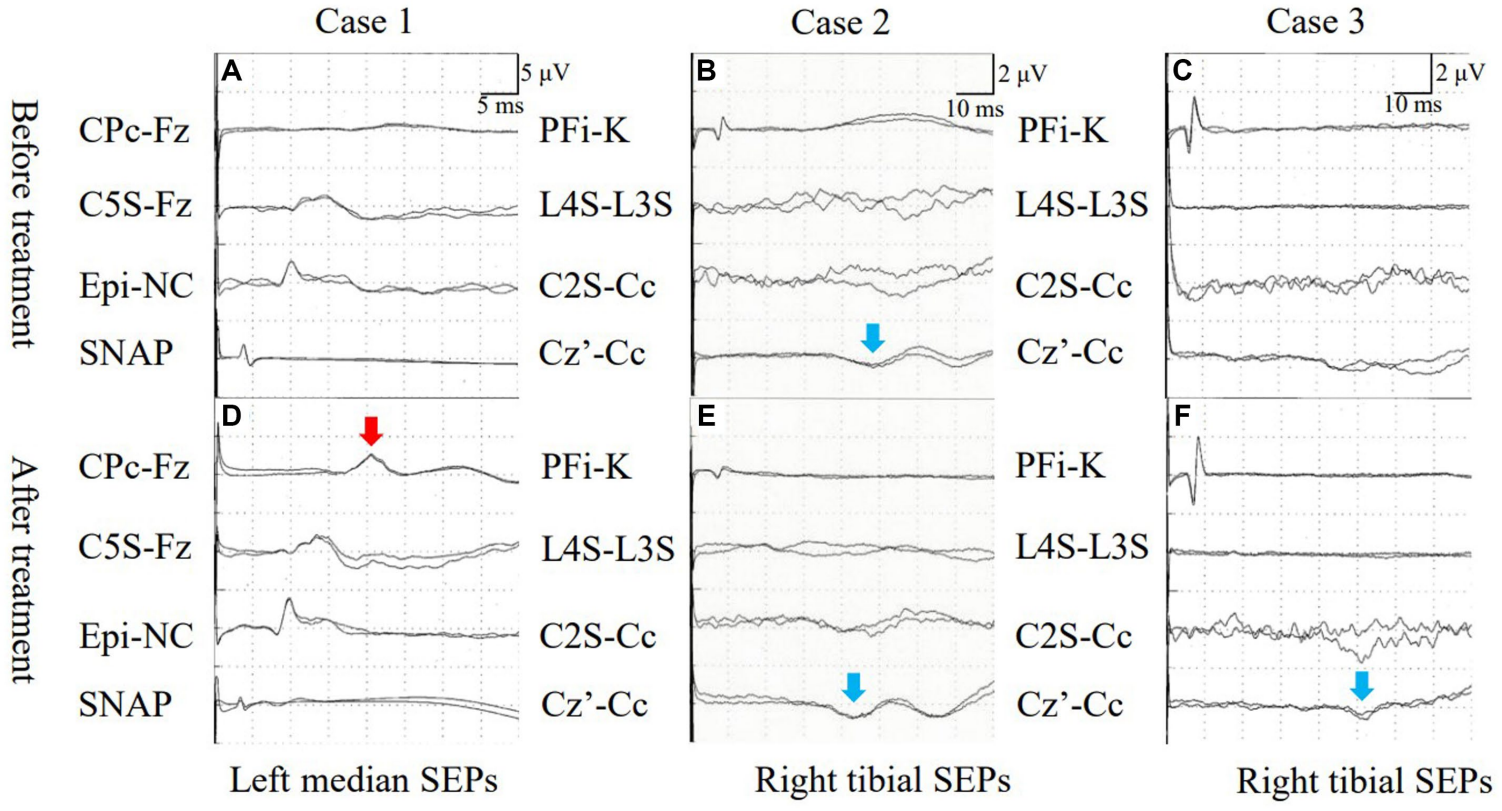
**(A)** Case 1: Median nerve SEPs demonstrated an absence of N20. **(B)** Case 2: Tibial nerve SEPs exhibited prolonged latency of P38. **(C)** Case 3: Tibial nerve SEPs indicated an absence of P38 activation (blue allow, **B**). **(D)** Case 1: Median nerve SEPs revealed N20 following treatment (red allow, **D**). **(E)** Case 2: Latency of tibial nerve SEPs decreased after treatment (blue allow, **E**). **(F)** Case 3: P38 activation was seen after treatment (blue allow, **F**). SEPs, somatosensory evoked potentials; SNAP, sensory nerve action potential.

A 45-year-old, male patient was admitted for numbness and dizziness. One month previously, he experienced a cough and fever and tested positive on nasopharyngeal polymerase chain reaction (PCR) for SARS-CoV-2. Two weeks after the onset of respiratory symptoms, he experienced numbness originating in the lower limbs and ascending to the chest and upper extremities. On admission, a neurological examination found weakness in the upper and lower extremities and spasticity in both lower limbs. His tendon reflexes were brisk, and Babinski sign were positive bilaterally. He had an unsteady gait and superficial sensory disturbances below the T4 level. Laboratory test results were normal. Autoantibodies, including anti-myelin oligodendrocyte glycoprotein (anti-MOG) antibody and anti-aquaporin 4 (anti-AQP4) antibody, were negative. A cerebrospinal fluid (CSF) analysis demonstrated cell count 6/μL (normal range: ≤ 5/μL) and positivity for oligoclonal bands (OCBs). A PCR test of CSF for SARS-CoV-2 PCR returned negative. No abnormal signals were detected on spinal MRI (Figure 1A). The results of nerve conduction studies were normal. The median nerve SEP demonstrated a reduced amplitude of N20 bilaterally (Figure 2A). Based on the neurological and SEP findings, the causative lesion was located within the spinal cord, and COVID-19-related myelitis was diagnosed. The patient received two cycles of intravenous methylprednisolone IVMP 1 g/day for three days followed by oral prednisolone 0.5 mg/kg of body weight, which improved his symptoms. Follow-up SEP findings showed normalization (Figure 2D), and MRI found no abnormalities (Figure 1E).

## Case 2

A previously healthy, 50-year-old, male patient received a SARS-CoV-2 vaccine 50 days before admission. Ten days after vaccination, a fever and malaise developed, and a PCR test of a nasopharyngeal sample returned positive for SARS-CoV-2. Although his fever improved, ten days after COVID-19 onset, he experienced a gait disturbance, bladder and bowel dysfunction, and sensory disturbances below the T10 level. On admission, a neurological examination demonstrated mild weakness with proximal predominance in the lower limbs. The left patellar tendon reflex was brisk while the other limb tendon reflexes were normal. Babinski sign was positive on the left side. The patient exhibited a slow, wide-based gait and was positive for Romberg’s sign and sensory disturbances below the L4 level. Laboratory test results, including those for the anti-AQP4 and anti-MOG antibodies, were normal. CSF analysis demonstrated cell count 6/μL and positivity for OCBs. Brain and spinal MRI demonstrated no abnormalities (Figure 1B). Nerve conduction study results were normal. The tibial nerve SEPs demonstrated a bilaterally reduced amplitude of N30 and P38 (Figure 2B). Myelitis was diagnosed on the basis of the neurological symptoms. Although only one cycle of IVMP was administered, the gait disturbance improved within two days, and the patient was negative for Romberg’s sign. Follow-up MRI demonstrated no abnormalities (Figure 1F), and the SEP findings demonstrated improved latency in P38 (Figure 2E).

## Case 3

A previously healthy, 44-year-old, male patient presenting with fever and cough tested positive on a nasopharyngeal antigen test for SARS-CoV-2. His respiratory symptoms improved a few days later, but dysgeusia developed. Ten days after COVID-19 onset, bladder dysfunction and periumbilical dysesthesia developed. Weakness and ascending numbness emerged in both lower extremities and gradually progressed, eventually producing a gait disturbance. On admission, the patient had lower extremity weakness with brisk reflexes and a wide-based gait. He was positive for Romberg’s sign and had sensory disturbances below the T7 level, pallhypesthesia, and impaired position sense in the lower extremities. Lhermitte’s sign was observed. Laboratory test results were normal. Antibodies, including serum anti-AQP4 and anti-MOG antibodies, were negative. CSF analysis demonstrated monocytes 8/μL and positivity for OCBs. Spinal MRI demonstrated normal signal intensity (Figures 1C,D). The results of nerve conduction studies were normal. Tibial nerve SEPs detected no P38 (Figure 2C). Immune-mediated myelitis associated with COVID-19 was diagnosed, and four cycles of IVMP were administered, followed by oral prednisolone 1 mg/kg and two cycles of intravenous immunoglobulin 0.4 g/kg for five days.

The neurological symptoms gradually improved, and a Romberg test was negative. Follow-up thoracic MRI found no abnormalities on day 90 after COVID-19 onset but found T2-weighted signal intensities in the dorsal columns from T1 to T10 without abnormal gadolinium enhancement on day 163 after onset (Figures 1G,H). Follow-up SEPs of the tibial nerve detected P38 with prolonged latency (51.3 ms) (Figure 2F).

## Discussion

We herein described three patients with subacute myelitis onset, no irregularities in the initial MRI findings, positivity for OCBs, and abnormal SEPs. Myelitis associated with COVID-19 was diagnosed on the basis of these findings together with negative results for various antibodies, including the anti-AQP4 and anti-MOG antibodies, and the fact that COVID-19 preceded the neurological symptoms. According to the WHO, an association between SARS-CoV-2 infection and myelitis/myelopathy is probable in the absence of any other explanatory pathogen or cause for the latter (6).

A clinical review of 43 patients with COVID-19-associated acute transverse myelitis estimated the incidence of COVID-19-associated myelitis at 0.5 per million (Román et al., 2021). The same study also reported that the main clinical manifestations were quadriplegia (58%) and paraplegia (42%). Additionally, spinal MRI results in 28 cases (70%) demonstrated longitudinally extensive acute transverse myelitis involving four or more spinal cord segments.

On the other hand, 15 cases of MRI-negative myelitis/myelopathy associated with COVID-19, including the present cases, have previously been reported (Table 1) (7–15) The latency period from the onset of SARS-CoV-2 infection to the first neurological manifestations was three weeks or less in nine patients (mean: 12.7 days) and two months or more in five patients (mean: three months). Ten patients presented with neurological symptoms with subacute onset, and five patients presented with symptoms with acute onset. Thirteen patients had paraplegia, indicating impairment at the level of the thoracic spinal cord. Two patients had tetraplegia, suggesting lesions in the cervical spinal cord. In most patients, CSF analysis revealed only a mildly elevated cell count. OCBs were detected in four of five patients. SEPs performed in four patients (12) revealed no abnormality.

**Table 1:** Summary of MRI-negative myelitis cases following COVID-19.

Author	Age (years) Sex	COVID-19 severity	Latency period	Onset	Symptoms	CSF	Management	Improvement
Case 1	45 M	Mild	14 days	S	Bl, Qu	Cells 7 /μL, OCB-positive	IVCS, PSL 0.5 mg/kg	Significant
Case 2	50 M	Mild	7 days	S	Bl, Pa, Ro	Cells 6 /μL, OCB-positive	IVCS, PSL 1 mg/kg	Significant
Case 3	44 M	Mild	10 days	S	Bl, Pa, Lh, Ro	Cells 8 /μL, OCB-positive	IVCS, IVIG PSL 1 mg/kg,	Significant
(7)	32 F	Mild	14 days	S	Bl, Pa, Lh	Cells 5/μL	IVCS, ACT, PSL 1 mg/kg	Significant
(8)	69 M	Asymptomatic	N/A	S	Bl, Pa	Protein 69.4 mg/dL	IVCS, IVIG	Absent
(8)	64 M	Mild	3–4 months	A	Bl, Pa, Lh	N/P	IVCS	Partial
(8)	64 F	Mild	5–6 months	A	Pa	N/P	N/A	N/A
(8)	58 F	Moderate	3 weeks	A	Pa	N/P	IVCS	Partial
(8)	63 M	Mild	2 months	S	Pa	N/P	N/A	N/A
(9, 10)	60 F	Mild	2 months	S	Bl, Pa, Lh	Cells 20 /μL, protein 81.6 mg/dL	ACT, PSL 60 mg, IVCS,PE	Significant
(11)	54 F	Mild	12 days	A	Bl, Pa,	Pleocytosis, OCB-negative	IVCS, IVIG	Partial
(12)	39 M	Mild	8 days	S	Bl, Pa, Lh	Pleocytosis	IVCS	Partial
(13)	22 F	Severe	15 days	A	Bl, Qu	Protein 53 mg/dL	Therapy for COVID-19 pneumonia	Improvement
(14)	63 M	Mild	12 days	S	Bl, Pa	Cells 16 /μL, protein 57.3 mg/dL	IVIG, IVCS	Partial
(15)	57 F	Mild	2 months	S	Bl, Pa	Elevated IgG	IVCS, PSL 60 mg	Significant

MRI, magnetic resonance imaging; COVID-19, coronavirus disease 2019; CSF, cerebrospinal fluid; M, male; F, female; A, acute onset; S, subacute onset; Bl, bladder and bowel dysfunction; Qu, quadriplegia; Pa, paraplegia; Lh, Lhermitte’s sign; Ro, Positive for Romberg’s sign; OCBs, oligoclonal bands; IVCS, intravenous corticosteroid; PSL, prednisolone; IVIG, intravenous immunoglobulin; ACT, anticoagulation therapy; PE, plasma exchange; IgG, immunoglobulin G; N/A, not available; N/P, not performed; mg, milligram; kg, kilogram; μl, microliter; dl, deciliter.

In the patient in Case 3, five months after the onset of neurological manifestations, areas of T2-weighted high signal intensities were detected on longitudinally extensive dorsal columns without abnormal gadolinium enhancement between T1 and T10. Memon et al. (9) reported that T2-weighted imaging demonstrated changes in the bilateral corticospinal tracts within the internal capsules extending to the cervical cord (C2-C6) without enhancement at four months after myelitis onset.

Of the previously reported 12 patients and the present, three patients, 12 received IVCS (intravenous corticosteroid), four received IVIG, one received plasma exchange, and two received anticoagulation therapy. In three, two, and one patient, oral prednisolone was administered at a relatively high starting dose of 1 mg/kg, 60 mg/kg, and 0.5 mg/kg of body weight, respectively. In the 13 patients receiving immunotherapy, six improved significantly, five improved partially, and one failed to improve. In the three patients in the present study, the mean duration from neurological symptom onset to immunotherapy was 30 days. However, the treatment efficacy was dramatic in all the patients. Zukic et al. (15) also reported an immediate response in their patients to IVMP and progressive improvement even though four months had elapsed since the onset of the neurological symptoms. Treatment is often delayed for COVID-19-associated myelitis because of the absence of abnormal MRI findings. However, the neurological symptoms may improve even with delayed treatment.

Although no hypothesis has been proposed to account for the pathophysiology of MRI-negative myelitis following COVID-19, the findings of the present study suggest several, potential mechanisms. The first hypothesis posits that the neural dysfunction is mediated by antibody binding. Sechi et al. (16) demonstrated that 10% of patients with MOG-associated myelitis had no abnormalities on their initial MRI, suggesting that MOG-associated myelitis with normal MRI findings might be associated with a dysfunction in MOG-IgG binding. In our study, all the patients were positive for OCBs, indicating that the neural dysfunction was mediated by the binding of unidentified antibodies.

A recent study using human brain organoids (HBOs) suggested that microglia play a key role in neurological complications following COVID-19. HBOs are self-organizing structures derived from human embryonic stem cells and containing neurons, astrocytes, and microglia. Kase et al. (17) reported that SARS-CoV-2 pseudotyped lentiviruses infected microglia in HBOs. Another study demonstrated that SARS-CoV-2 infection activated microglia in HBOs, leading to the elimination of synapses. (18) The findings of these studies suggest that infection in microglia leads to their activation, which in turn results in neurological deficits.

Only a few studies have focused on the association between MRI abnormalities and microglial activation. A study comparing pathological and MRI findings in multiple sclerosis (MS) found microglial activation in regions that appeared normal on MRI. (19). This finding suggests that MRI may not be sensitive enough to detect microglial activation.

In the patient in Case 3, MRI abnormalities appeared five months after the onset of neurological symptoms. Memon et al. (9) similarly reported MRI signal changes occurring four months after onset and speculated that these signal changes might reflect intense axonal degeneration without demyelination. In the patient in Case 3, the neurological symptoms progressively improved about one year after onset. However, Wallerian degeneration cannot explain the reversible pathophysiology. To the best of our knowledge, no hypothesis can, as of yet, adequately account for the delayed MRI findings. However, we speculate that this finding may reflect reactive changes caused by an abnormal immune response possibly instigated by SARS-CoV-2 infection.

The present study has some limitations. First, the sample pool included only three patients. However, these patients had similar, clinical characteristics. Further investigation and statistical analysis involving more cases are needed. Second, although the patients were monitored for relatively long periods, these periods varied significantly between individuals. Further longitudinal study is therefore needed. Last, the present study lacked pathological examination and relied largely on pathophysiological assessments in previous studies.

## Conclusion

The present report described three cases of MRI-negative myelitis following COVID-19. The absence of MRI abnormalities may result in delayed diagnosis and treatment. In such situations, SEPs can play a vital role in confirming spinal cord localization. The pathogenic mechanism in the present cases was considered heterogeneous and included an abnormal immune response and microglial activity specific to COVID-19. The subacute progression was characteristic of MRI-negative COVID-19-associated myelitis. Even delayed initiation of immunosuppressive therapy may significantly improve the clinical course.

## Data availability statement

The original contributions presented in the study are included in the article/supplementary material, further inquiries can be directed to the corresponding author.

## Ethics statement

The studies involving humans were approved by Tokyo Metropolitan Neurological Hospital. The studies were conducted in accordance with the local legislation and institutional requirements. The participants provided their written informed consent to participate in this study. Written informed consent was obtained from the patients for the publication of this case report.

## Author contributions

KK: Writing – original draft, Writing – review & editing. RS: Writing – original draft, Writing – review & editing. YS: Writing – original draft, Writing – review & editing. NM: Writing – review & editing. ST: Writing – review & editing. TS: Writing – review & editing. KT: Writing – review & editing.
